# Feasibility of Hepatic Fat Quantification Using Proton Density Fat Fraction by Multi-Echo Chemical-Shift-Encoded MRI at 7T

**DOI:** 10.3389/fphy.2021.665562

**Published:** 2021-05-07

**Authors:** Radim Kořínek, Lorenz Pfleger, Korbinian Eckstein, Hannes Beiglböck, Simon Daniel Robinson, Michael Krebs, Siegfried Trattnig, Zenon Starčuk, Martin Krššák

**Affiliations:** 1 Magnetic Resonance group, Institute of Scientific Instruments of the Czech Academy of Sciences, Brno, Czechia; 2 Division of Endocrinology and Metabolism, Department of Medicine III, Medical University of Vienna, Vienna, Austria; 3 Department of Biomedical Imaging and Image-Guided Therapy, High-Field Magnetic Resonance Centre, Medical University of Vienna, Vienna, Austria; 4 Christian Doppler Laboratory for Clinical Molecular Imaging, CD Laboratory for Clinical Molecular MR Imaging (MOLIMA), Medical University of Vienna, Vienna, Austria

**Keywords:** CSE-MRI, ultra-high magnetic field, 7T, feasibility, liver, PDFF

## Abstract

Fat fraction quantification and assessment of its distribution in the hepatic tissue become more important with the growing epidemic of obesity, and the increasing prevalence of diabetes mellitus type 2 and non-alcoholic fatty liver disease. At 3Tesla, the multi-echo, chemical-shift-encoded magnetic resonance imaging (CSE-MRI)-based acquisition allows the measurement of proton density fat-fraction (PDFF) even in clinical protocols. Further improvements in SNR can be achieved by the use of phased array coils and increased static magnetic field. The purpose of the study is to evaluate the feasibility of PDFF imaging using a multi-echo CSE-MRI technique at ultra-high magnetic field (7Tesla). Thirteen volunteers (M/F) with a broad range of age, body mass index, and hepatic PDFF were measured at 3 and 7T by multi-gradient-echo MRI and single-voxel spectroscopy MRS. All measurements were performed in breath-hold (exhalation); the MRI protocols were optimized for a short measurement time, thus minimizing motion-related problems. 7T data were processed off-line using Matlab® (MRI:multi-gradient-echo) and jMRUI (MRS), respectively. For quantitative validation of the PDFF results, a similar protocol was performed at 3T, including on-line data processing provided by the system manufacturer, and correlation analyses between 7 and 3T data were performed off-line. The multi-echo CSE-MRI measurements at 7T with a phased-array coil configuration and an optimal post-processing yielded liver volume coverage ranging from 30 to 90% for high- and low-BMI subjects, respectively. PDFFs ranged between 1 and 20%. We found significant correlations between 7T MRI and -MRS measurements (R^2^ ≅ 0.97; *p <* 0.005), and between MRI-PDFF at 7T and 3T fields (R^2^ ≅ 0.94; *p* < 0.005) in the evaluated volumes. Based on the measurements and analyses performed, the multi-echo CSE-MRI method using a 32-channel coil at 7T showed its aptitude for MRI-based quantitation of PDFF in the investigated volumes. The results are the first step toward qMRI of the whole liver at 7T with further improvements in hardware.

## Introduction

The importance of fat fraction quantification and distribution in the human tissue is growing with the epidemic of obesity, and the increasing prevalence of diabetes mellitus type 2 (T2DM) [1, 2] and non-alcoholic fatty liver disease (NAFLD) [3–5].

Modern 3T clinical MR systems, providing high signal-to-noise ratio (SNR) and high resolution, allow the measurement of tissue proton density fat fraction (PDFF) [6, 7] even in routine clinical protocols. Multi-echo data is required to achieve accurate fat quantification based on Chemical Shift Encoded Magnetic Resonance Imaging (CSE-MRI) [8–13]. To determine of accurate PDFF distribution in the inner organs, high image resolution is required. Then, however, the SNR is reduced and subsequent data processing, in which the PDFF is calculated for each image voxel/pixel, is adversely affected. In principle, the SNR can be increased by the use of phased array RF (multi-channel) coils [14–16] and/or increased static magnetic field [17–19], both having some practical limits and incurring higher costs of hardware [20]. Recent installations of ultra-high-field (UHF) MR systems (7T or more), along with improvements in RF hardware and acquisition methods, have clearly demonstrated superior data quality for neuro-and musculoskeletal imaging [21, 22]. Nevertheless, problems remain; in particular, the abdominal region is affected by water-fat displacement and stronger susceptibility artifacts. Moreover, at high fields, the chemical shifts between the water peak and the multiple spectral peaks in the fat signal are increased. To minimize chemical shift displacement, strong gradients and large acquisition bandwidths must be used, causing SNR reduction and potentially substantial eddy currents [23, 24]. The other UHF effects are a prolongation of relaxation time *T*_1_ and a shortening of *T*_2_ and ${\text{T}}_{2}^{*}$ relaxation times [25–27]. An important parameter in UHF MR for patient safety is the specific absorption rate (SAR), which, in principle, increases quadratically with *B_0_* field, but the specific spatial pattern depends in a complex way on the B_1_ frequency and a variety of factors, including the subject [28, 29]. These factors are a problem mainly with whole-body or large-volume coverage coils, such as birdcage, saddle, and TEM coils. A related UHF-MR problem is inhomogeneity of the excitation RF-field $\left(B_{1}^{+}\right)$, which leads to inhomogeneous excitation and errors in quantitative imaging if not properly addressed. To overcome the problems of heterogeneous SAR, excitation, and detection sensitivity, dedicated multi-channel RF coils in transmit and receive modes [30–33] have been used and field-specific adaptations to MR acquisition protocols have been implemented [34, 35].

CSE-MRI-based quantification of PDFF is a fast and reliable way to determine the distribution of fat in a tissue. This approach, proposed by Dixon [36] in 1984, has undergone considerable changes and evolution [8, 9, 13, 37, 38]. These various changes are frequently called “Dixon” methods even for multi-echo approaches; however, the term “CSE-MRI” is usually used for advanced methods that include a multi-echo acquisition scheme. Generally, the signal model representing the behavior of a vector of magnetization during the measurement sequence is crucial for the calculation of correct MRI-PDFF values. Due to the complex lipid spectrum [6, 39, 40], which contains several spectral lines in the frequency range of ~4.5 ppm (main peak at 1.3 ppm -CH_2_-), the signal model must contain prior knowledge about the spectral position and the relative intensity of each peak. A further common problem of all “Dixon”/CSE-MRI water-fat separation methods is field map (B_0_) estimation [11, 41]. At higher static magnetic fields, larger local magnetic field gradients result in more phase overflows of 2π in complex images compared to those at lower fields. The occurrence of these phase wraps may lead to water-fat swaps in the calculated MRI-PDFF maps, necessitating the application of phaseunwrapping algorithms. Several approaches have addressed this problem successfully [37, 41]. In hepatic iron overload, especially at excessive concentration, the tissue signal is significantly dephased, and hence, ${\text{T}}_{2}^{*}$ relaxation is distinctly shortened; thus, the ${\text{R}}_{2}^{*}=1/{\text{T}}_{2}^{*}$ must be included in the signal model [8]; otherwise, substantial errors in PDFF estimation [42, 43] may occur.

In fact, the current clinical 3T protocols for PDFF measurements collect data in low resolution due to the requirement for a short measurement time (breath-hold) and a good SNR. The full coverage of the abdominal space is standard at 3T; however, the lower resolution smooths the information about the fat or iron distribution in the liver. This is a potential problem for the detailed study of fat distribution in the liver. Higher resolution that prolongs measurement time influences other measuring parameters (TE, TR, BW, and many others), and mainly deteriorates SNR in acquired data, which leads to noisy parametric maps (PDFF, R2*). Generally, quantitative magnetic resonance imaging (qMRI) is a crucial component of the many therapies and diagnostic [44], and provides the relatively stable and reproducible results [45]. The using of high field potentially yield benefit in the form of higher SNR compare to a low field, and it can improve the qMRI [46]. We have to note at the outset that due to incomparable coil configuration, the quality of images is not compared (SNR) in the study (it would not be objective); and the main focus is on the comparison of the quantitative results from both magnetic field, 3 and 7T. This study is the first step in exploring the possibilities of abdominal quantitative MRI (qMRI) that could provide improvement in diagnostic accuracy for a wide range of chronic liver diseases due to higher sensitivity at 7T in combination with the appropriate hardware equipment.

The purpose of this study was to assess the feasibility of proton density fat fraction (PDFF) quantification using multi-echo MRI at 7T with a 32-channel phase-array coil without B_1_ shimming. To demonstrate the potential value of the method at UHF with the best possible available hardware configuration at our institution (at the time of the study), PDFF derived from 7T MRI measurement (MRI-PDFF) was compared to PDFF determined by 7T MRS (MRS-PDFF) and to gold-standard [47, 48] multi-echo MRI-based 3T measurements [49, 50] on the same group of subjects.

## Materials and Methods

### Subjects

Thirteen subjects (4f/9m; age, 44.7 ± 14.7 years; body mass index (BMI) 25.6 ± 4.7 kg.m^–2^; mean ± SD) participated in this study. Volunteers were recruited based on the hepatic PDFF values obtained in previous studies [51–53] to cover a broad range of PDFFs (0–20%) without a focus on their respective health status or diagnosis. The group comprised six lean volunteers (BMI = 21.7 ± 1.9 kg.m^–2^) and seven volunteers with high BMI (BMI = 29.6 ± 2.7 kg.m^–2^).

#### Ethics Statement

The study was approved by the local ethics committee. Informed consent was obtained from all individual participants included in the study.

### Acquisition and Reconstruction

#### 7T Measurements

At 7T (MAGNETOM, Siemens Healthineers, Erlangen, Germany), a phased array receive/transmit surface (32-channel) coil (Cardiac Transceiver Array RF Coil, MRI.TOOLS GmbH) was used. MR image data were acquired with an accelerated 3D-SPGR sequence with bipolar readout gradients with the following parameters: field of view (FOV) = 38.0 cm × 33.2 cm; acquisition bandwidth (BW) = 1,395 Hz/pixel; repetition time (*T*_R_) = 9.6ms; flip angle (FA) = 4°; 32 slices (slice gap of 20%); acquisition matrix size in-plane = 256 × 224 pixels; voxel size 0.74 × 0.74 × 4 mm^3^; and six echoes with an equidistant echo spacing of Δ*T*_E_ = 1.81ms (the shortest possible), where the first *T*_E_ = 1.45ms, and acquisition time *T*_A_ = 11.8 s, with a GRAPPA [54] acceleration factor of 8. Multi-channel multi-echo data were combined with the scanner image reconstructor using ASPIRE [55] and the PDFF maps were generated using the Graph-Cut approach [37] in a MATLAB® toolbox [56, 57], including the prior knowledge of the multi-frequency fat spectrum [39]. Single-voxel proton spectroscopic measurements were performed using a modified STEAM sequence [27] with *T*_R_ = 5 s, with echo times *T*_E_ = 6, 12, and 20ms, *T*_M_ = 10ms, and a voxel size of 30 × 30 × 30 mm^3^. Due to a relatively narrow frequency bandwidth of the excitation pulse, the measurement was repeated with the same parameters, but with the excitation frequency (delta frequency) offset by –3.4 ppm from the water frequency (4.7 ppm) to fully cover the frequency band around the main fat resonances at 1.3 ppm (CH_3_). The acquired spectra were evaluated in jMRUI [58] with the AMARES fitting algorithm [59, 60] with a prior knowledge of the fat spectral components [39]. The MRS-based PDFF was calculated from *T*_2_-corrected spectra as the ratio of the estimated relative proton density of mobile lipids to the sum of the estimated relative proton densities of mobile water and mobile lipids.

#### 3T Measurements

At 3T (Trio/PrismaFit, Siemens Healthineers, Erlangen, Germany), a combination of phased-array abdominal (18 channels) and spinal (32-channel) receiver coils and a whole-body transmit coil supplied by the MR-system manufacturer was used for data acquisition. MR image data were acquired by an accelerated 3D-SPGR (Spoiled Gradient Echo) [61] sequence with unipolar readout gradients with the following parameters: FOV = 38.0 cm × 31.4 cm; BW = 1.040 Hz/pixel; *T*_R_ = 9.32ms; FA = 3° (to minimize *T*_1_ effects); 48 slices (slice gap of 20%); acquisition matrix size in-plane = 160 × 104 pixels (interpolated to 320 × 264 pixels); voxel size 1.2 × 1.2 × 3.5 mm^3^; and six echoes with an equidistant echo spacing of Δ*T*_E_ = 1.31ms, where the first *T*_E_ = 1.23ms, *T*_A_ = 6.9 s, and a CAIPIRINHA [62, 63] acceleration factor of 4 (2 × 2). The MRI protocol contains the online water-fat separation provided by the system manufacturer, which allows direct visualization of PDFF maps immediately after the acquisition. Those maps were used in our evaluation. The implemented online water-fat separation is certified for clinical use; therefore, such reconstructed data (PDFF maps) were used as the 3T MRI reference. Moreover, supplementary PDFF maps were reconstructed from 3T MRI data using the same approach as in 7T to allow a comparison of the same processing algorithm applied to data from different magnetic fields.

### Volume of Interest Selection

The VOI for the 7T MRI-PDFF (similar or almost identical volumes compare to MRS) data analysis and 7T MRS data acquisition was placed in a homogeneous hepatic tissue carefully chosen to avoid contamination from liver vessels and subcutaneous tissue, but in the vicinity of the multi-channel coil, thus ensuring the best possible signal-to-noise. Selection of a VOI (similar size as 7T case) in the same position was attempted on the 3T PDFF maps.

### Effective Liver Volume Coverage

To evaluate the range of coil combinations used at 7T, the term “effective liver volume coverage” was introduced where the liver volume at 7T was compared with the “true liver volume.” The “true liver volume” was estimated from 3T images for each subject where the full coverage of the abdominal region (not only the liver) was expected (100% coverage); the liver segmentation was performed manually. At 7T, the image noise thresholding segmentations on measured data (echo images of each subject for the longest TE) were performed to identify the background noise regions in the images, and these masks were applied to the measured data to achieve masked images. Subsequently, the liver was manually segmented from the masked images.

### Statistical Analyses

The reconstructed 3T- and 7T-MRI-PDFF data where displayed in a box-and-whisker diagram to show the distribution of fat within the investigated VOIs for each subject. To prove the relations between 7T-MRI, 3T-MRI and 7T-MRS PDFF measurements, linear regression and Bland-Altman analyses were performed. All statistical tests were performed in MATLAB (MathWorks, Natick, MA, USA).

## Results

**Figure 1:**
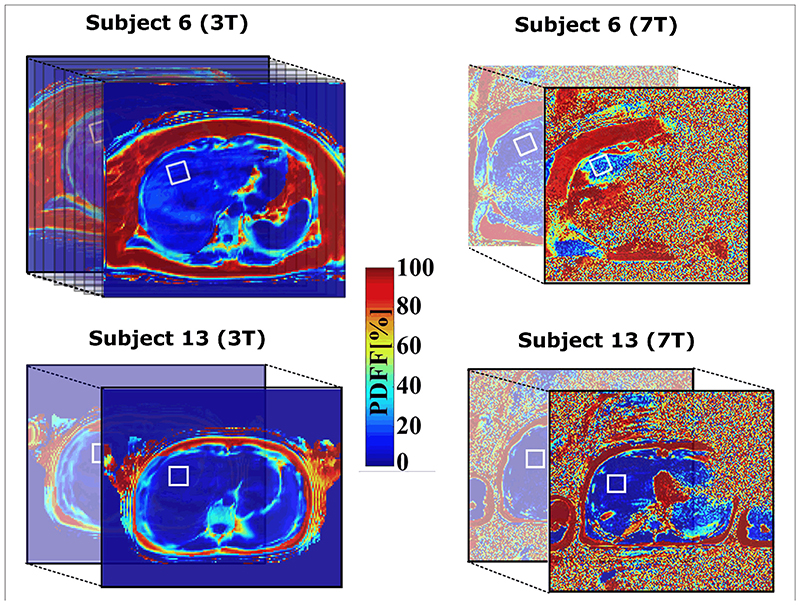
Positions of VOIs in the cranial section of the liver of subjects S6 and S13 with lowest and highest BMI (within the experimental group of our subjects), respectively. The investigated region is depicted by white solid boxes across the volume. The 7T data are noisy due to higher resolution and bandwidth per pixel compare to 3T.

**Figure 2:**
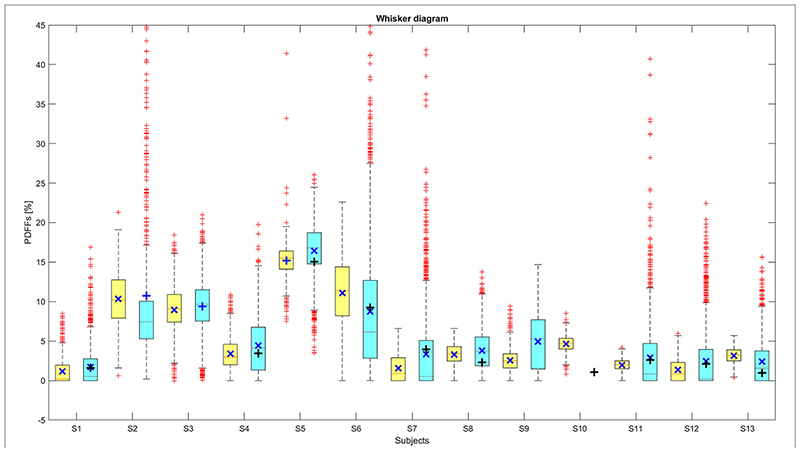
The distributions of MRI-PDFF for individual subjects at 3T (yellow boxes) and 7T (blue boxes). The related 7T-MRS-PDFF values are depicted by black solid horizontal crosses. The red horizontal lines in the bars are the medians and the dark blue solid oblique crosses are the mean values of these distributions. The bottom and top of the boxes represent the 25th and 75th percentile (Q1 and Q3 quartiles) of MRI-PDFF distributions. The ends (black horizontal lines) of the extended whiskers (dashed black lines) define upper and lower extremes (± 2.7σ ≈ 99.3% coverage).

Examples of the acquired MRI-PDFF volumes for two subjects with different body sizes and composition, and with high (S6) and low (S3) BMI, at 3 and 7T, are shown in Figure 1 (and related figures of PDFFs with corresponding 7T anatomical images in Supplementary Material 1). The position of the spectroscopic volume of interest (VOI) and the ROI used for the comparison is delineated for each subject by the white box in the respective PDFF map; the calculated field maps are shown in Supplementary Material 2. The effective liver volume coverage for MRI-PDFF showed 100% of the liver volume, as well as the whole abdomen for all subjects at 3T. In the case of the 32-channel RF coil used at 7T, the effective liver volume coverage varied from ~30 to 90% based on body size and composition. Liver coverage was highest in the subjects with low BMI and smaller body (torso) size (example in Figure 1 S13, Supplementary Figures 1, 3 in Supplementary Material 1). A case of a subject with very high BMI and non-optimal body size is shown in Figure 1 S6 and Supplementary Figures 1, 2 in Supplementary Material 1. The whiskers diagrams (Figure 2) show the distributions of MRI-PDFF within the VOI and the related MRS-PDFF values acquired from similar or almost identical volumes.

**Figure 3:**
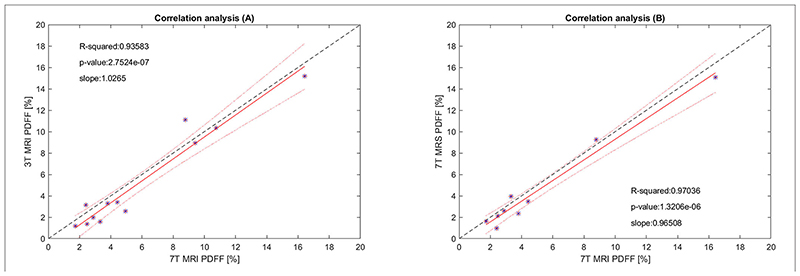
The correlation analysis of **(A)** 7T-MRI-PDFF vs. 3T-MRI-PDFF, and **(B)** 7T-MRI-PDFF vs. 7T-MRS-PDFF The red line represents the linear fit of the values, and 95% confidence intervals for the slope of the lines are depicted by red dotted lines (upper and lower bounds). The black dashed line corresponds to a perfect match. *Its data point.

**Figure 4:**
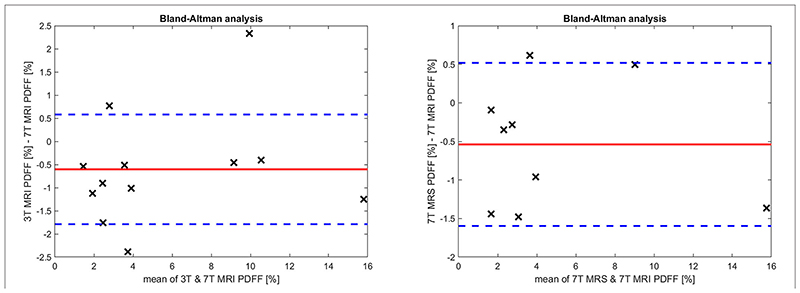
The Bland Altman analysis for (left) 3T and 7T MRI, and (right) 7T MRI and MRS measurements. The red line depicts the mean difference (bias) of all measurements, and the blue dotted lines represent the 95% confidence intervals for the mean.

The correlation analyses for the data presented in Figure 2 are shown in Figure 3, where the 7T-MRI-PDFFs are compared with (Figure 3A) 3T-MRI, and (Figure 3B) 7T-MRS PDFF values. In the both cases A and B (Figure 3), high *R* ^2^-values with low *p*-values, indicating strong agreement between the 3T and 7T measurements, were observed. The calculated *R* ^2^-values (with p-values) of A and B (Figure 3) cases were 0.936 (p ≈ 2.75·10^–7^; 7T MRI vs. 3T MRI) and 0.970 (p ≈ 1.32·10^–6^; 7T MRI vs. 7T MRS), respectively; in addition, the slopes were calculated. Then, the Bland-Altman (BA) analyses of the previous (A) and (B) cases were also performed, which can be seen in Figure 4. In the first case, the BA plot shows a mean difference (bias) of ≈-0.60% and upper and lower confidence intervals (CI of 95%) of 0.58 and -1.79%, respectively. In the second case (Figure 4 on the right), there was a mean difference (bias) of ≈-0.54% and upper and lower confidence intervals (CI of 95%) of 0.52 and –1.60%, respectively. The extended boxplots and related analyses of PDFF maps that were calculated by the offline toolbox using Graph-Cut algorithm/approach from 3T-MRI data are given in Supplementary Material 3.

In subject S2, the 7T MRS measurement was not performed because of health problems (cramps) not related to the measurement. The reconstructed (7T) MRI-PDFF maps (mainly the volume of interest) from S10 were affected by strong water-fat swaps that could not be removed by changing the parameters of the algorithm used, and therefore, the results were excluded. The 7T spectra from subjects S3 and S9 were not included in the analysis due to movement artifacts in the data.

## Discussion

In our study, we tested the feasibility of MRI-PDFF assessment at 7T. Even-though MRS was considered for the “reference method” in hepatic fat content quantification, recent developments in MRI-PDFF at 3T have substantially improved the accuracy of the approach and made it the method of choice for hepatic fat content quantification [47, 48] in clinical settings.

The results depicted in Figure 2 demonstrate an agreement between the MRI-PDFF distributions from 3T and 7T measurements in the investigated volumes. In most cases, the spectroscopic values were within the 25th and the 75th percentile of MRI-PDFF. In only one case was the MRS value not in this interval (Figure 2), probably due to subtle motion of the subject between the MRS and MRI measurements. Another possible deteriorating effect is intra-voxel inhomogeneity, due to movement-related changes in local B_0_, which can lead to an improper estimation of PDFF. In 7T, MRI-DFF distributions show a larger range of values, and the number of outliers (red crosses, Figure 2) is higher than at 3T. This can be caused by the inhomogeneity of ${\text{B}}_{1}^{–}$ over the FOV if the investigated volume is relatively distant from the coil surface, and hence, the water-fat separation process is more prone to errors due to lower SNR.

Correlation analyses confirm good agreement between MRI-PDFF 7T and 3T measurements in the investigated volumes. The level of agreement is attributable to measurement conditions, such as the measurement sequences (minimizing the acquisition time), patient measurement management (short time period between the 3T and 7T measurements–not more than 1 h), and exhalation breath-hold. From our practical experience, the bias in BA-plots appears relatively low, given that we are comparing measurements at different magnetic field. Although initial preliminary studies concerning hepatic liver fat quantitation by MRI at 7T have already been published [64, 65], in this study, a larger number of subjects was investigated, the distribution of MRI-PDFF in the investigated volumes was analyzed and compared to already established 7T MRS, and 3T MRI measurements were performed.

## Limitations

At the 7T, due to the unavailability of whole-body ^1^H transmit RF hardware and B1 shimming equipment, precise positioning of the multichannel Tx/Rx surface coil was necessary, and the quality of data and liver volume coverage also depended on the subject/patient body size and composition. In subjects with a low BMI, the liver volume coverage was sufficient and water-fat separation provided PDFF maps without water/fat swaps within the liver tissue. In subjects with a high BMI and body size, the PDFF maps were affected by water-fat swaps and determining the optimal parameters for the water-fat separation process was more complex. Nevertheless, the Graph-Cut approach applied here is flexible enough with regard to the input parameters (such as the regularization parameter, spatial subsampling for field map estimation, the range of field map, and many others), and, in many cases, a sufficient solution was found (i.e., the PDFF maps of liver cross-section or at least the VOI not affected by water/fat swaps). Furthermore, it is important to note that the use of ASPIRE for the combination of the multi-channel phase data ensured the correct input data for the used GC approach.

At this moment, PDFF-MRI at 7T with the phased-array Tx/Rx coil used does not provide satisfactory coverage of the whole liver for patients with a higher BMI (Figure 1, Supplementary Materials 1, 2), and does not provide any significant advantage over PDFF-MRI at 3T. The effective liver coverage is based on the patient body composition and the resulting electrical properties of a measured subject. We have to admit that the effective liver coverage dropping to 30% in some cases is a significant drawback of our 7T configuration compared to current 3T measurements where full liver coverage is not an issue, and that appears to be the main limitation of the study. The other potential limitations are minimal echo time and echo spacing, but in fact it can be a problem especially in approaches where are estimated individual fat components [66, 67]. In our case, we have the prior knowledge of fat spectral model. However, there is strong potential to improve the quality of whole-liver imaging at 7T. Subjects with a high BMI frequently accompanied by oversized abdominal organs results in practical problems, such as B_0_ shimming, RF power settings, and the coverage of the whole liver volume. The quality of 7T data could be improved using interactive *B*_1_ shimming [68, 69], and liver coverage could be increased by the use of a volume body coil [70] in combination with an array coil [71], but neither of these was available for this study. Nevertheless, there are several possible pulse sequence options that may improve data quality in the future: Shorter echo spacing (use of shorter excitation pulses) and implementation of CAIPIRINHA acceleration can provide a significant reduction of acquisition time compared to GRAPPA, which was the only available parallel imaging option on our 7T system.

## Conclusion

Our results confirm the feasibility of hepatic fat content quantification by MRI-PDFF based on a multi-gradient-echo acquisition method at ultra-high field (7T) using a 32-channel Tx/Rx array coil. In addition to a coil configuration and other hardware equipment, the success of fat quantification using MRI-PDFF is based on the water-fat separation algorithm, including prior knowledge of the fat spectral model. In the presence of rapid field changes at 7T, a robust solution that avoids or at least minimizes water/fat swaps in the reconstructed MRI-PDFF maps (most importantly in the regions of interest) is required. The advanced coil configuration with the further envisioned hardware improvement will provide the opening for further improvement of whole-abdomen imaging and liver fat quantification for patients with a higher BMI.

## Supplementary Material

Supplementary Material

## Data Availability

The raw data supporting the conclusions of this article will be made available by the authors, without undue reservation.
