# Supplementary material for: Feasibility of Hepatic Fat Quantification Using Proton Density Fat Fraction by Multi-Echo Chemical-Shift-Encoded MRI at 7T
Source: Front Phys. Author manuscript; Available in PMC 2021 Nov 29. (PMC7612048; doi:10.3389/fphy.2021.665562)
Supplement: Supplementary Material [file EMS138821-supplement-Supplementary_Material.zip › Data_Sheet_1_Feasibility of Hepatic Fat Quantification Using Proton Density Fat Fraction by Multi-Echo Chemical-Shift-Encoded MRI at 7T.docx]

Supplementary Material (Supplement 1)


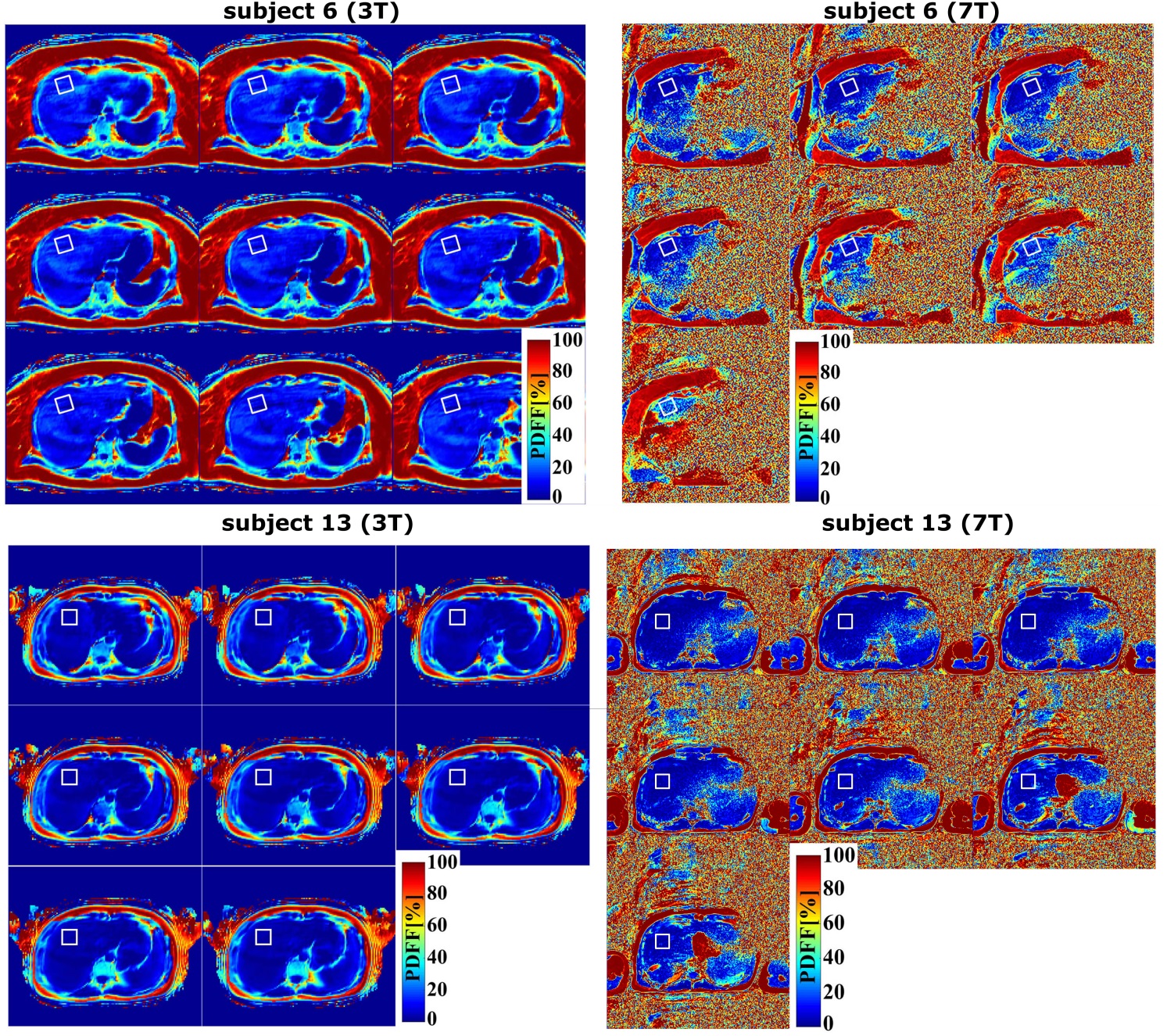


**Figure Supp1-1.** Examples (subjects S6 and S13) of MRI-PDFF maps of slices cutting the investigated volume of interest (VOI) at 3T and 7T fields. The position of the investigated volume that corresponds to the largest axial cross-section in the cranial section of the liver (same/similar as MRS) is depicted by white solid boxes in MRI-PDFF maps.


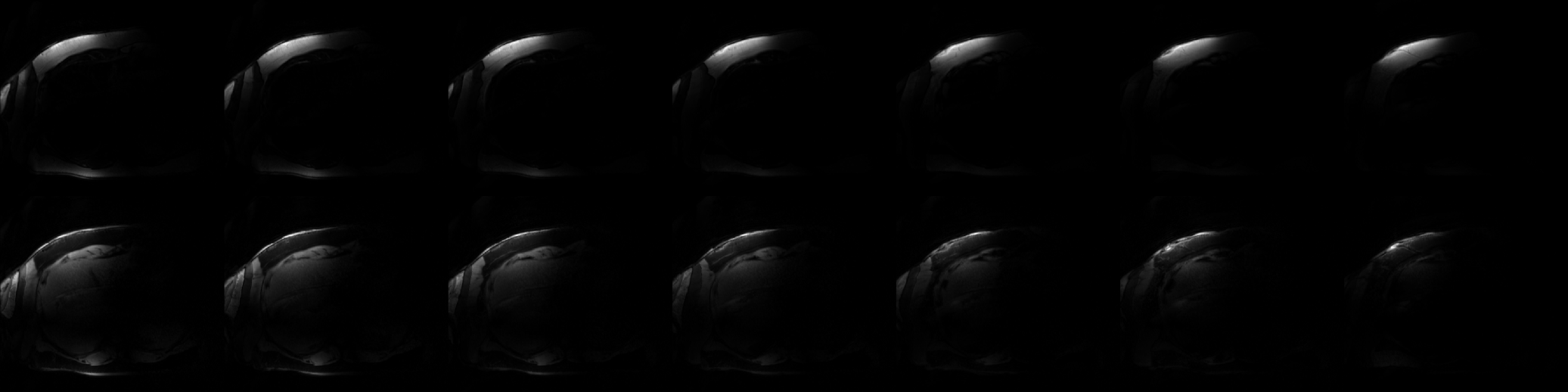


**Figure Supp1-2.** The anatomical reconstructed fat and water images of subject S6 at 7T field (related PDFF maps in Figure Supp1-1.).


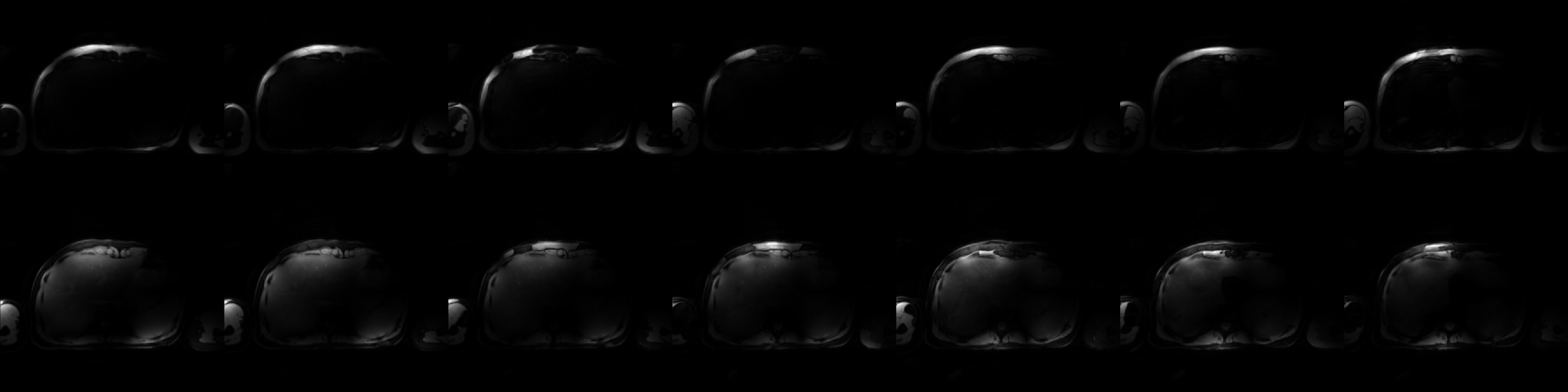


**Figure Supp1-3.** The anatomical reconstructed fat and water images of subject S13 at 7T field (related PDFF maps in Figure Supp1-1.).
