# Supplementary material for: Feasibility of Hepatic Fat Quantification Using Proton Density Fat Fraction by Multi-Echo Chemical-Shift-Encoded MRI at 7T
Source: Front Phys. Author manuscript; Available in PMC 2021 Nov 29. (PMC7612048; doi:10.3389/fphy.2021.665562)
Supplement: Supplementary Material [file EMS138821-supplement-Supplementary_Material.zip › Data_Sheet_2_Feasibility of Hepatic Fat Quantification Using Proton Density Fat Fraction by Multi-Echo Chemical-Shift-Encoded MRI at 7T.docx]

Supplementary Material (Supplement 2)

A
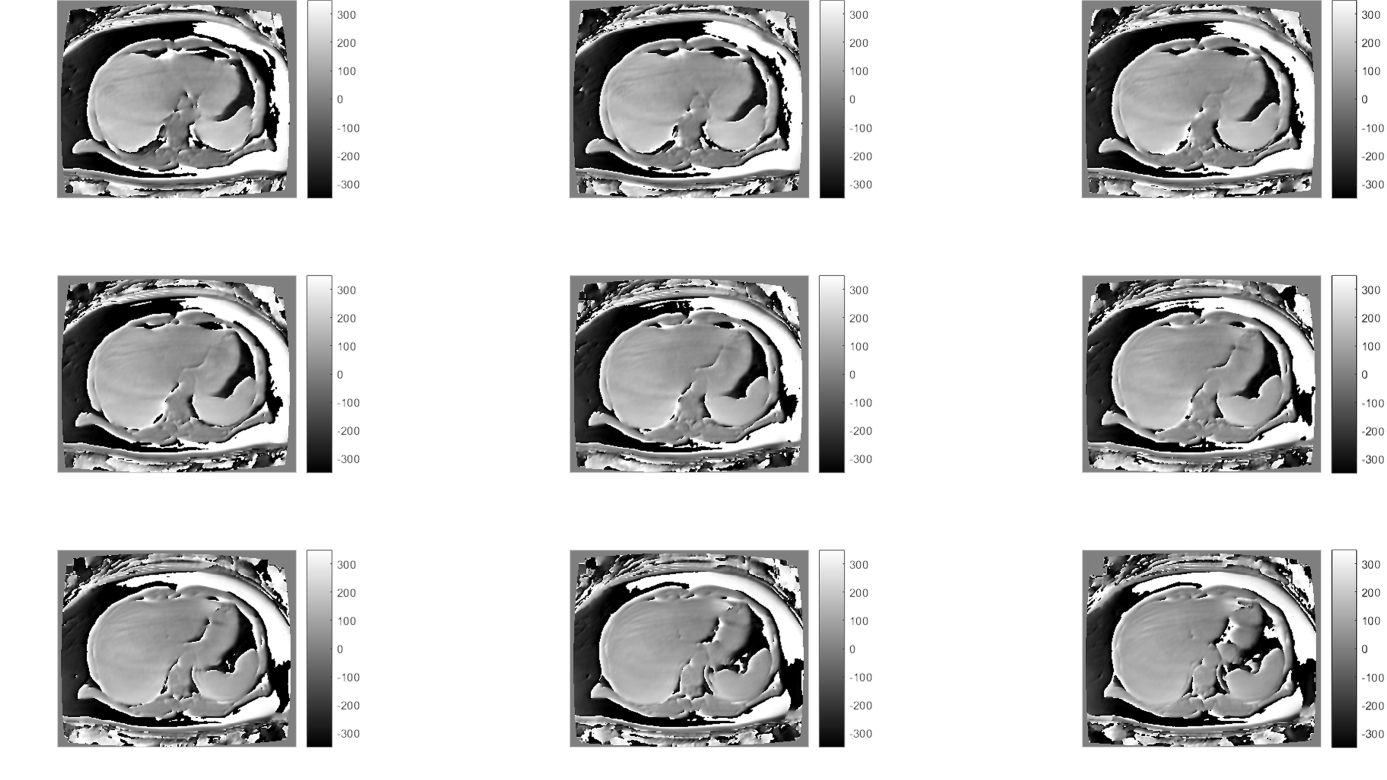
B
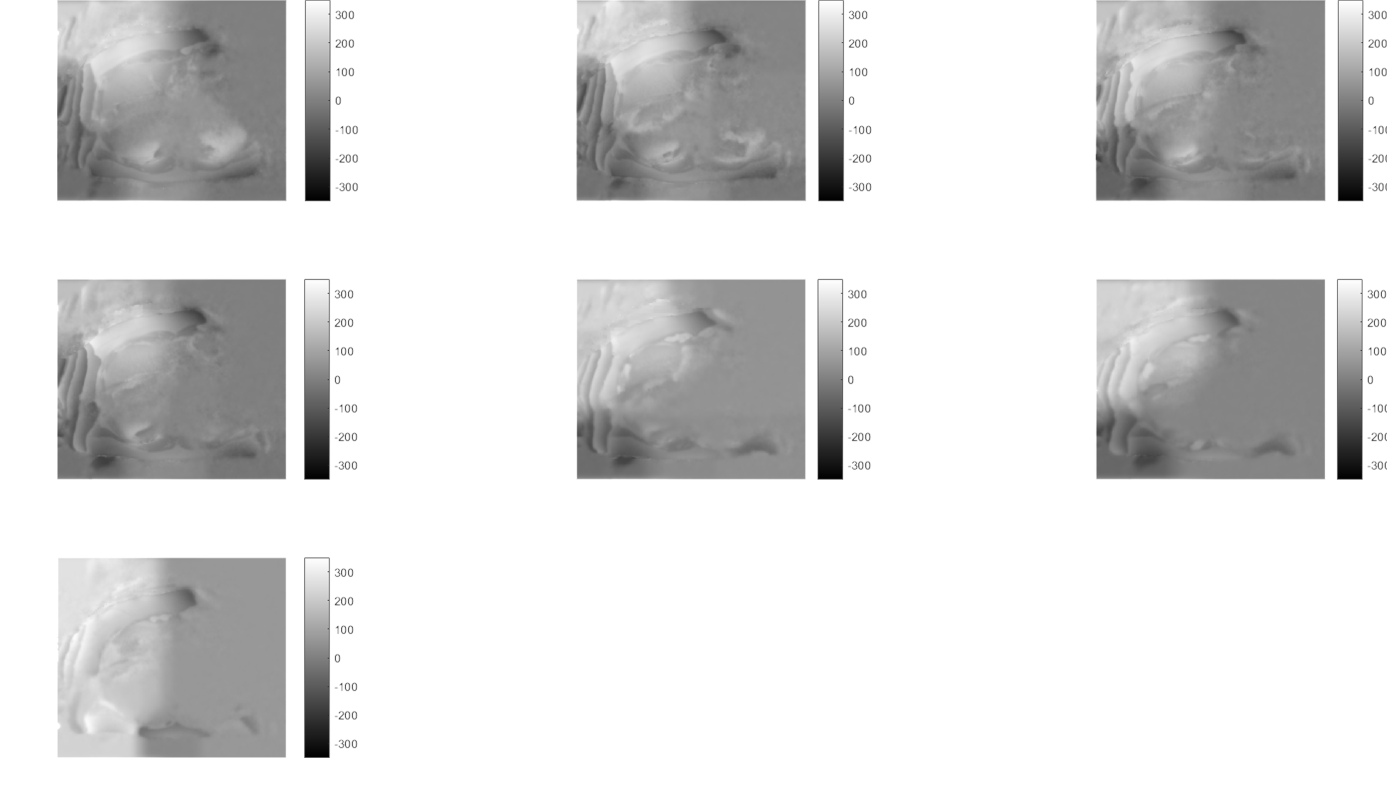


**Figure Supp2-1.** Field maps in Hz for A) 3T and B) 7T measurements of subject 6.

A
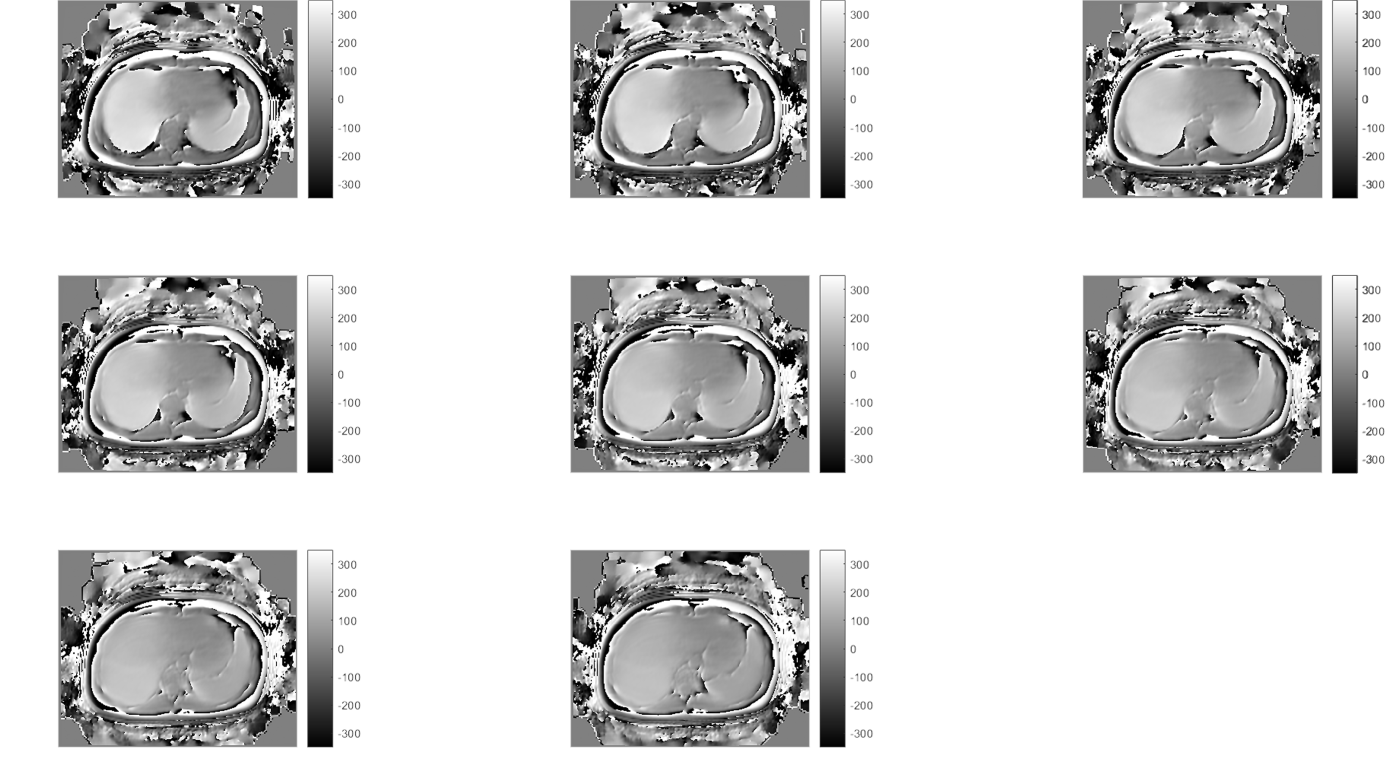
B
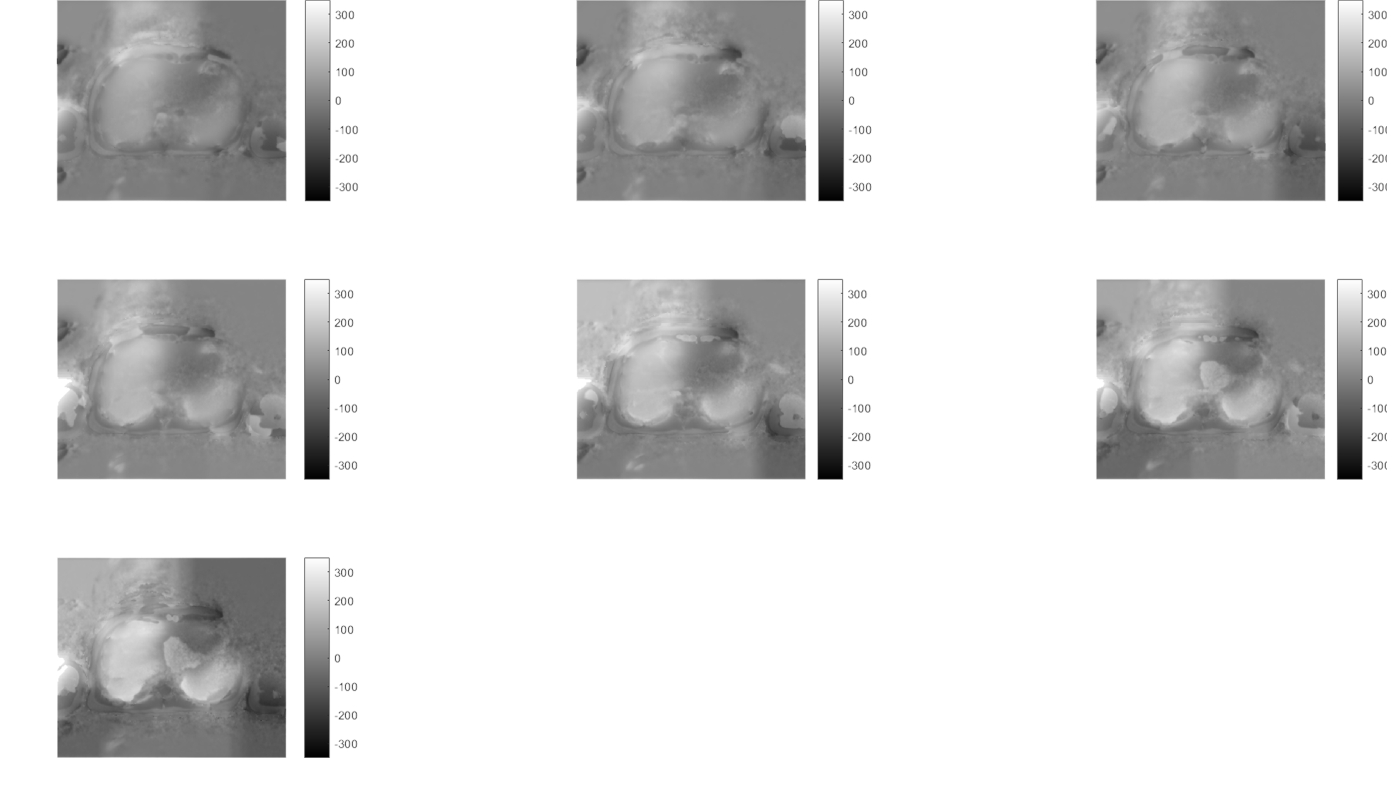


**Figure Supp2-2.** Field maps in Hz for A) 3T and B) 7T measurements of subject 13.
