# Supplementary material for: Feasibility of Hepatic Fat Quantification Using Proton Density Fat Fraction by Multi-Echo Chemical-Shift-Encoded MRI at 7T
Source: Front Phys. Author manuscript; Available in PMC 2021 Nov 29. (PMC7612048; doi:10.3389/fphy.2021.665562)
Supplement: Supplementary Material [file EMS138821-supplement-Supplementary_Material.zip › Data_Sheet_3_Feasibility of Hepatic Fat Quantification Using Proton Density Fat Fraction by Multi-Echo Chemical-Shift-Encoded MRI at 7T.docx]

Supplementary Material (Supplement 3)


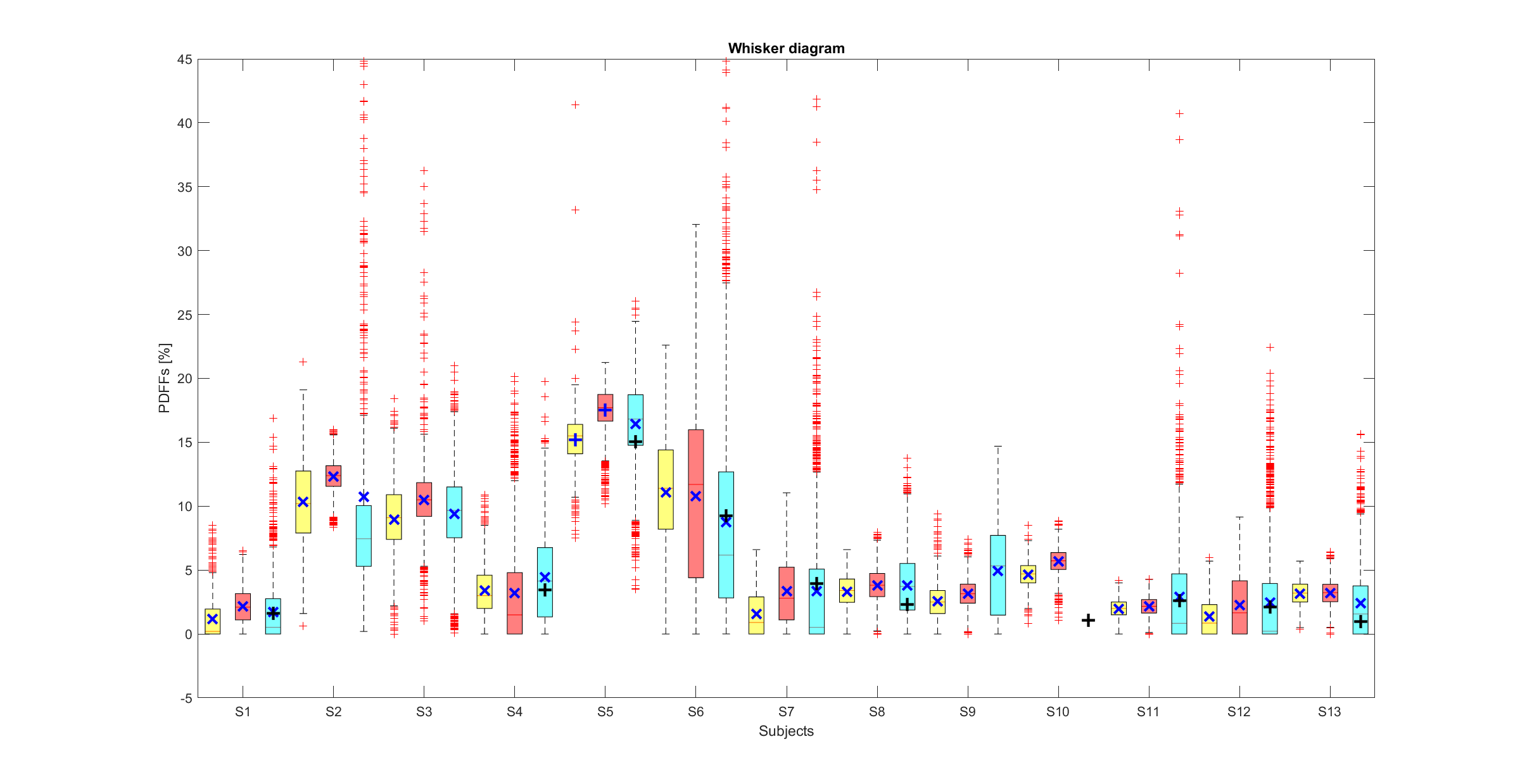
**Figure Supp3-1**. The distributions of MRI-PDFF for individual subjects at 3T (yellow boxes – online reconstruction; red boxes – Graph Cut algorithm) and 7T (blue boxes) fields. The related 7T-MRS-PDFF values are depicted by black solid horizontal crosses. The red horizontal lines in the bars are the medians and the dark blue solid oblique crosses are the mean values of these distributions. The bottom and top of the boxes represent the 25th and 75th percentile (Q1 and Q3 quartiles) of MRI-PDFF distributions. The ends (black horizontal lines) of the extended whiskers (dashed black lines) define upper and lower extremes (± 2.7σ ≈ 99.3% coverage).


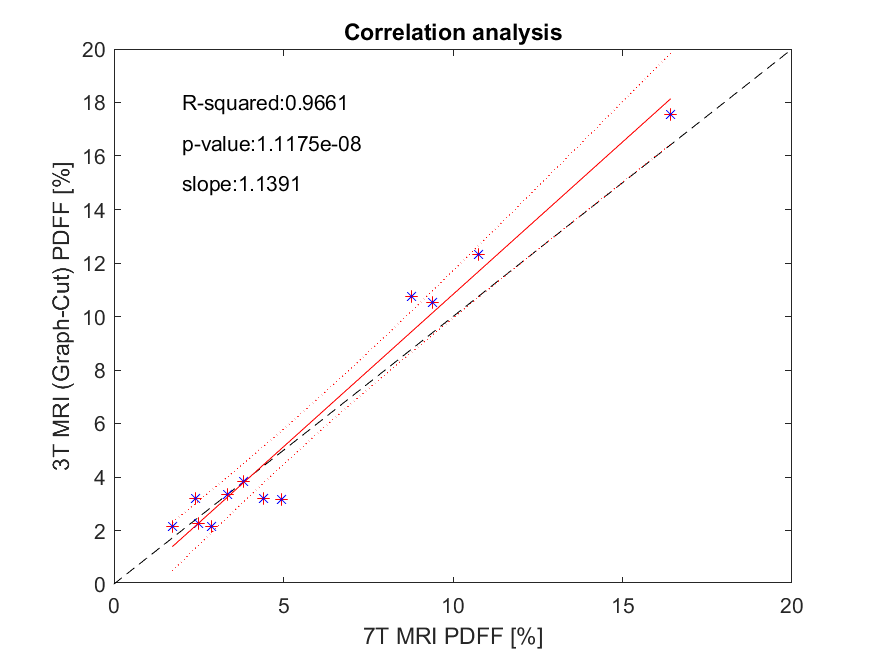


**Figure Supp3-2.** The correlation analysis of 7T-MRI-PDFF vs 3T-MRI-PDFF (Graph-Cut). The red line represents the linear fit of the values, and 95% confidence interval for the slope of the line are depicted by red dotted-lines (upper and lower bounds). The black dashed line corresponds to perfect match


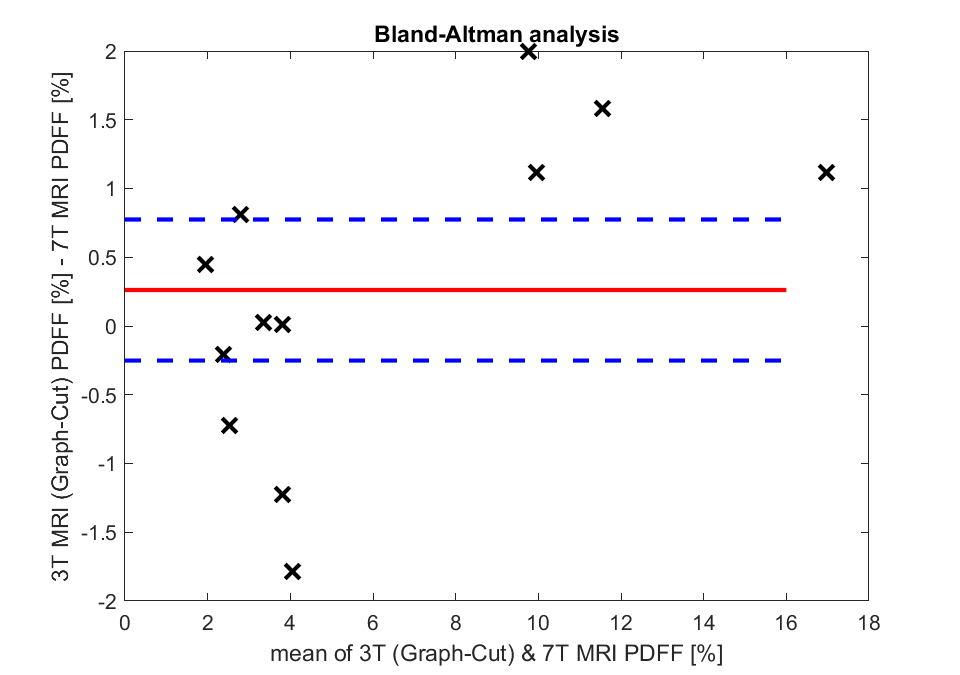


**Figure Supp3-3.** The Bland Altman analysis for 3T (Graph-Cut) and 7T MRI measurements. The red line depicts the mean defference (bias) of all measurements, and the blue-dotted lines represents the 95% confidence interval for the mean.
